# TET3 Mediates 5hmC Level and Promotes Tumorigenesis by Activating AMPK Pathway in Papillary Thyroid Cancer

**DOI:** 10.1155/2022/2658727

**Published:** 2022-06-15

**Authors:** Jiadong Chi, Wei Zhang, Yigong Li, Jie Zhao, Xiangqian Zheng, Ming Gao

**Affiliations:** ^1^Department of Thyroid and Neck Tumor, Tianjin Medical University Cancer Institute and Hospital, National Clinical Research Center for Cancer, Key Laboratory of Cancer Prevention and Therapy, Tianjin's Clinical Research Center for Cancer, Tianjin 300060, China; ^2^School of Medicine, Nankai University, Tianjin 300071, China; ^3^Department of Breast and Thyroid Surgery, Tianjin Union Medical Center, No. 190 Jieyuan Road, Hongqiao District, Tianjin 300121, China; ^4^Department of Orthopedics, Tianjin Hospital, Tianjin University, Tianjin 300211, China

## Abstract

Thyroid cancer is the most common endocrine malignant tumor. The accurate risk stratification and prognosis assessment is particularly important for patients with thyroid cancer, which can reduce the tumor recurrence rate, morbidity, and mortality effectively. DNA methylation is one of the most widely studied epigenetic modifications. Many studies have shown that 5hmC-mediated demethylation played an important role in tumors. The hydroxylation of 5mC is catalyzed by ten-eleven translocation dioxygenase (TET). In this study, we first found that the abnormal expression of 5hmC was closely related to microcarcinoma, multifocal, extraglandular invasion and lymph node metastasis of thyroid carcinoma. Then, we identified TET3 was differentially expressed in thyroid cancers and normal tissues from the TET family. TET3 can promote the proliferation, migration, and invasion of thyroid cancer. TET3-mediated 5hmC can regulate the transcription of AMPK pathway-related genes to activate the AMPK pathway and autophagy and therefore promote PTC proliferation. These findings provide a preclinical rationale for the design of novel therapeutic strategies for this target to improve the clinical outcome of patients with PTC.

## 1. Introduction

Thyroid cancer is the most common endocrine malignant tumor. In recent decades, the incidence of differentiated thyroid cancer has increased worldwide, including papillary thyroid cancer (PTC), follicular thyroid cancer (FTC), and Hurthle cell cancer. At present, surgery is still the main treatment for papillary thyroid carcinoma. After surgical resection, most of the patients with papillary thyroid carcinoma have a good prognosis. However, after the initial treatment, the possibility of recurrence is still high. The follow-up studies of the patients found that the 10-year recurrence rate of differentiated thyroid cancer is about 20%, and the 30-year recurrence rate is about 30% [1–3]. Therefore, the accurate risk stratification and prognosis assessment is very important for patients with thyroid cancer, which can reduce the tumor recurrence rate, morbidity, and mortality effectively.

DNA methylation is one of the most widely studied epigenetic modifications. Many studies have confirmed that DNA methylation plays a key role under different conditions [4–6]. DNA methylation in vertebrates usually occurs at the CpG (cytosine-phosphate-guanine) site, which is in the DNA sequence that follows cytosine followed by guanine. 5-methylcytosine (5mC) is one of the derivatives of cytosine. Other derivatives include 5-hydroxymethylcytosine (5hmC), 5-formyl cytosine (5fC), and 5-carboxyl cytosine (5caC). The most common methylation product is 5mC [7], and the 5hmC produced by hydroxylation of 5mC is the main product of DNA demethylation, which plays an important role in the elimination of methyl [8, 9] and is closely related to tumorigenesis [10–13]. Many studies have shown that 5hmC-mediated demethylation could eliminate methylation status, resulting in the treatment of tumors [14–18]. The hydroxylation of 5mC is catalyzed by ten-eleven translocation dioxygenase (TET) [9, 19, 20]. In many tumors, the loss of 5hmC is often associated with abnormal regulation of TET [21, 22].

AMP-activated protein kinases (AMPK) are highly conserved from yeast to plants and animals and play a key role in regulating energy balance and nutrient metabolism [23, 24]. As a metabolic sensor, AMPK allows adaptive changes in metabolic coordination, cell growth, apoptosis, and autophagy [25]. It has been reported that the activation of AMPK can inhibit tumor growth by blocking the MAPK and PI3K-AKT signaling pathways [26, 27].

Autophagy is a catabolic process in which the autophagy lysosome degrades most of the cytoplasmic contents [28]. Autophagy is usually activated in the context of undernutrition but is also involved in many physiological processes, including development, differentiation, and many human cancers [26, 27]. It can be induced under stressful conditions such as low-energy charge or lack of necessary nutrients [29]. Notably, autophagy can be promoted by AMPK [30].

In this study, we first performed 5hmC immunohistochemical staining on paraffin specimens of PTC and found that the abnormal expression of 5hmC was closely related to the clinicopathologic characteristics of thyroid carcinoma including microcarcinoma, multifocal, extraglandular invasion, and lymph node metastasis. We identified TET3 was differentially expressed in thyroid cancers and normal tissues from the TET family. TET3 can promote the proliferation, migration, and invasion of thyroid cancer. TET3-mediated 5hmC can regulate the transcription of AMPK pathway-related genes to activate the AMPK pathway and autophagy, therefore promoting PTC proliferation. Our study identifies a novel carcinogenesis mechanism of thyroid cancer caused by TET3-mediated DNA methylation. It provides a new biomarker for the prognosis and treatment of thyroid cancer.

## 2. Materials and Methods

### 2.1. Cell Culture

Human thyroid cancer cell lines TPC-1 and BCPAP were purchased from American Type Culture Collection. RPMI1640 medium (Gibco) containing 10% fetal bovine serum (volume fraction) and 1% cyan/streptomycin (Solarbio) was cultured in a constant temperature incubator with 5% CO_2_ at 37°C. Cell growth was observed under the microscope.

### 2.2. Immunohistochemical Staining

In this study, 50 patients with papillary thyroid carcinoma were selected by random sampling from January 2016 to December 2016 in the Department of Thyroid and Neck Oncology, Tianjin Medical University Cancer Institute and Hospital. The research was performed with the approval of the Ethics Committee of Tianjin Medical University Cancer Institute and Hospital. Informed consent was obtained for experimentation with human subjects. Dissected tumor tissues and adjacent normal tissues were preserved in 10% formalin for24 h, dehydrated through xylenes and alcohols, and embedded in paraffin. Sections were cut at 5 *μ*m. Immunohistochemistry was performed according to standard immunohistochemistry protocol. Signal was visualized with DAB Substrate Kit (MaiXin Bio). Images were taken with Leica DM3000 Microscope.

### 2.3. Dot Blot

DNA extraction and dot-blot analysis were performed as previously described [31]. In brief, total DNA was extracted using an Animal Tissues/Cells Genomic DNA Extraction Kit (Solarbio), following the manufacturer's protocol. DNA samples were loaded onto Amersham Hybond-N + membrane (GE Healthcare, Chicago, IL) and cross-linked to the membrane with UV radiation. Then, the membrane was blocked with 5% nonfat dry milk (in PBST) for 1-2 h, incubated with a specific anti-5hmC antibody (Abcam, ab214728, 1 : 2000) overnight at 4°C followed byHRP-conjugated anti-rabbit IgG (Cell Signaling Technology) for 1 h at room temperature, and then developed with Thermo ECL SuperSignal Western Blotting Detection Reagent (Thermo Fisher Scientific, Waltham, MA).

### 2.4. Quantitative Real-Time PCR (qRT-PCR)

mRNA extraction and qRT-PCR were performed as previously described [32]. Total RNA was isolated from fresh-frozen thyroid cancer tissues and thyroid cancer cell lines using TRIzol reagent (Invitrogen, Carlsbad, USA) and reverse-transcribed to cDNA using PrimeScript RT Master (Takara, Kyoto, Japan). Real-time PCR was performed by using SYBR Premix Ex Taq II (Takara, Kyoto, Japan) and specific primers. The primer sequences are listed in Supplementary Table 1.

### 2.5. Western Blotting

Western blotting was performed as previously described [33]. Cells were lysed with RIPA lysis buffer (Solarbio), and protein concentrations were determined by BCA. The samples were then mixed by SDS-PAGE. Gel proteins were transferred to PVDF membranes (Beyotime) using standard Bio-Rad wet transfer apparatus, blocked with 5% skim milk (Solarbio), and incubated with primary antibodies overnight at 4°C. The next day, PVDF membranes were incubated with secondary antibodies, and chemiluminescent detection was performed using the Western Blotting Detection Kit ECL (Human IgG) (Solarbio).

### 2.6. Immunofluorescence

Cell slides were prepared before the experiment. The slides of attached cells were fixed with 4% paraformaldehyde for 20 minutes, permeated with 0.5% Triton X-100 for 15 minutes, and sealed with 5% BSA for 1 hour at room temperature. Each slide was added with enough diluted primary antibody and placed overnight in a humidifier at 4°C. Next day, the slides were washed 3 times with TBST, and a diluted fluorescent secondary antibody (Beyotime) was added, followed by incubation at room temperature for 1 hour in the dark. The slides were washed 3 times using TBST and sealed using 5 *μ*L of antifade mounting medium with DAPI (Invitrogen). Images were acquired under a fluorescence microscope.

### 2.7. Colony Formation

The cells were seeded into 6-well plates with 1 × 10^6^ cells in each well. When the cell fusion rate reached 80%, the cells were digested and 1 × 10^3^ cells from each group were seeded into 6-well plates for further culture for 10 days. Then, the clones were fixed with paraformaldehyde and dyed with 0.4% crystal violet. The number of cell clones in each group was calculated under the microscope.

### 2.8. Cell Viability Assay

The logarithmic growth phase cells were inoculated into 96-well plates at the rate of 2 × 10^3^with 100 *μ*L complete medium (1640 + 10% FBS) in each well and incubated in 37°C, 5% CO_2_ incubator with corresponding time. Then added 100 *μ*L 1640 medium and 10 *μ*L CCK-8 reagent (Solarbio) to each well and incubated for another 2 hours. OD value was detected by 450 nm wavelength microplate analyzer.

### 2.9. Transwell Assay

When the cell fusion rate reached 80%, the cells were collected and inoculated into the upper layer of Matrigel-coated transwell chamber (Corning) with 1 × 10^4^ cells/chamber. Culture medium without fetal bovine serum was added for culture, and normal cell culture medium was added into the lower layer. After continued culture for 24 hours, the cells were fixed with paraformaldehyde and stained with 0.1% crystal violet. Under the microscope, 5 fields were randomly selected from each group and stained cells were counted.

### 2.10. Wound-Healing Assay

The cells were seeded into 6-well plates with 1 × 10^6^ cells in each well. When the degree of cell fusion reached 90%, the cells were scratched at the bottom with sterilized 10 *μ*L spear tip and washed with PBS to remove the exfoliated cells. The width of the scratch was measured at 0 hour and 24 hours under inverted microscope. Scratch closure rate (%) = (0-hour scratch area—24 hours scratch area)/0-hour scratch area × 100%.

### 2.11. Cell Cycle Analysis

Cells in logarithmic growth state were digested and fixed with 75% ethanol overnight. 1 mL working solution containing PI (final concentration is 50 g/mL) (Solarbio) and RNA enzyme (final concentration is 50 g/mL) (Solarbio) was added to the stain for 30 minutes, and cell cycle was detected by flow cytometry.

### 2.12. Cell Transfection

Small interfering RNAs (siRNAs) were directly synthesized (GenePharma). The cells were seeded into 6-well plates with 1 × 10^6^ cells in each well. When the degree of cell fusion reached 50%, siRNAs were transfected into cells using Lipofectamine 2000 (Invitrogen) according to the manufacturer's instructions. 48 hours later, the cells were harvested for further experiments.

### 2.13. Statistical Analysis

SPSS 19.0 statistical software was used to analyze all experimental data, which were presented as mean ± standard deviation (−x ± S). Student's *t* test (two-sided) was used to calculate the significance of differences between groups. The results between the two groups were tested by chi-square test. *χ*^2^ test analyses were performed to determine the expression difference of 5hmC in normal tissues and tumor tissues as well as the relationship between the 5hmC expression and clinicopathologic characteristics in PTC. *P* value <0.05 was considered statistically significant.

## 3. Results

### 3.1. Study on the Relationship between Demethylation and Papillary Thyroid Carcinoma

In this study, 50 patients with papillary thyroid carcinoma were selected by random sampling from January 2016 to December 2016 in the Department of Thyroid and Neck Oncology, Tianjin Medical University Cancer Institute and Hospital, using SPSS statistical software (Table 1).

**Figure 1:**
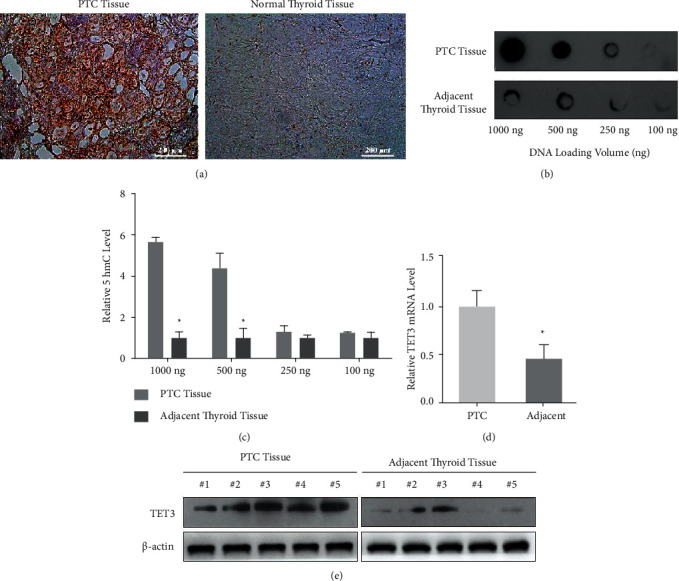
(a) The results of hydroxymethylation 5hmC staining in clinical specimens of papillary thyroid carcinoma showed that 5hmC was expressed in papillary thyroid carcinoma and normal thyroid tissues adjacent to the cancer, and the positive expression was brown (scale bar = 200 *μ*m); (b) the results of genomic DNA 5hmC dot blot hybridization (dot blot) of papillary thyroid carcinoma;. (c) statistical analysis of genomic DNA 5hmC dot blot hybridization (dot blot) in papillary thyroid carcinoma; (d) real-time PCR was used to detect the expression of TET3 mRNA in papillary thyroid carcinoma and normal thyroid tissues; (e) western blot was used to detect the expression of TET3 in papillary thyroid carcinoma and adjacent normal thyroid samples. Sample size = 50 *μ*g, internal reference: *β*-actin.

The corresponding pathological sections of 50 patients performed immunohistochemistry staining with 5hmC antibody, and the results showed that the expression of 5hmC in PTC tissues was increased (Figure 1(a) and Table 2). *χ*^2^ tests analysis was used to analyze the statistical results of staining and the clinicopathological features of PTC. The results showed that the factors associated with positive 5hmC hydroxymethylation were microcarcinoma, multifocal, extraglandular invasion, and lymph node metastasis, while the unrelated factors were sex, age, and lateral grade (Table 3).

To further prove the correlation between genomic DNA demethylation and clinicopathological features of thyroid carcinoma, samples of 20 patients with papillary thyroid carcinoma were selected using a random number method. Genomic DNA was extracted from tumor tissues and normal thyroid adjacent to cancer for 5hmC hydroxymethylation dot blot hybridization (dot blot). The results showed that the content of 5hmC in papillary thyroid carcinoma was significantly increased (Figures 1(b) and 1(c)).

The occurrence and development of papillary thyroid carcinoma are closely related to the demethylation level of tumor DNA. In cytosine demethylation metabolism, demethylase TET family is a kind of oxygenase that can catalyze the metabolism of 5-methylcytosine (5mC) to 5hmC, mainly including TET1, TET2, and TET3.

To study the mechanism of elevated 5hmC level in papillary thyroid carcinoma DNA, we extracted RNA, from papillary thyroid carcinoma and adjacent normal tissues, and detected the expression levels of TET3 in these 20 samples by real-time quantitative PCR (real-time PCR). The results showed that the expression of TET3 in papillary thyroid carcinoma was significantly higher than that in normal paracancerous tissues (Figure 1(d)) while the expression of TET1 and TET2 showed no difference in cancer tissues and normal tissues (Supplementary Figure 1A).

We further selected the cancer foci and adjacent normal tissues of 5 patients with papillary thyroid carcinoma using a random number method and verified the expression of TET3 is higher in cancer foci (Figure 1(e)). It is suggested that the abnormal expression of DNA demethylase TET3 may play an important role in the occurrence and development of papillary thyroid carcinoma by regulating the metabolism of 5mC to 5hmC in DNA.

### 3.2. Inhibition of TET Expression Significantly Decreased the Level of Methylation and the Ability of Proliferation, Migration, and Invasion of PTC Cells

**Figure 2:**
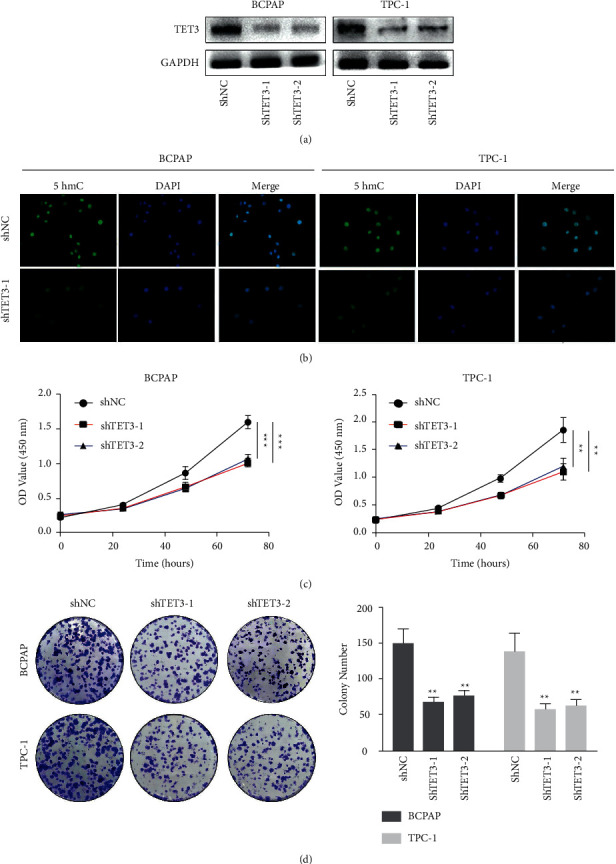
(a) Western blot verified the expression level of TET3 was decreased with shTET3 intervention in BCPAP and TPC-1 cells; (b) the results of immunofluorescence showed that inhibiting the expression of TET3 significantly decreased the 5hmC level of papillary thyroid carcinoma cell lines BCPAP and TPC-1. Upper panel: 5hmC staining (green); lower panel: DAPI staining (blue), superimposed with 5hmC staining; (c) CCK-8 assays and (d) colony formation assays showed that TET3 knockdown significantly suppressed cell proliferation in BCPAP and TPC-1 cells.

We transfected two independent short hairpin RNA (shRNA) targeting TET3 into BCPAP and TPC-1, two classic papillary thyroid carcinoma cell lines, to inhibit the expression of TET3, and then verified the repression of TET3 expression by Western blot (Figure 2(a)). The main function of DNA demethylase TET3 is to catalyze the metabolism of 5mC to 5hmC. In this study, immunofluorescence was used to detect the level of hydroxymethylation modification of BCPAP and TPC-1 cell lines with or without shTET3 intervention. The results showed that after TET3 knockdown, the level of hydroxymethylation modification was significantly lower than that in the control group (Figure 2(b)). In addition, CCK-8 analysis (Figure 2(c)) and clone formation analysis (Figure 2(d)) showed that the low expression of TET3 in BCPAP and TPC-1 cells significantly inhibited cell proliferation.

**Figure 3:**
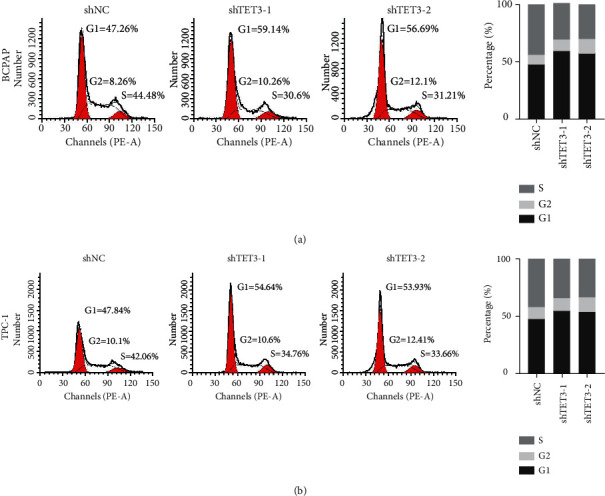
Knockdown of TET3 significantly induced cell-cycle G1/S arrest and summarized data regarding the cell numbers at each cell-cycle phase after TET3 knockdown in BCPAP (a) and TPC-1 (b) cells.

**Figure 4:**
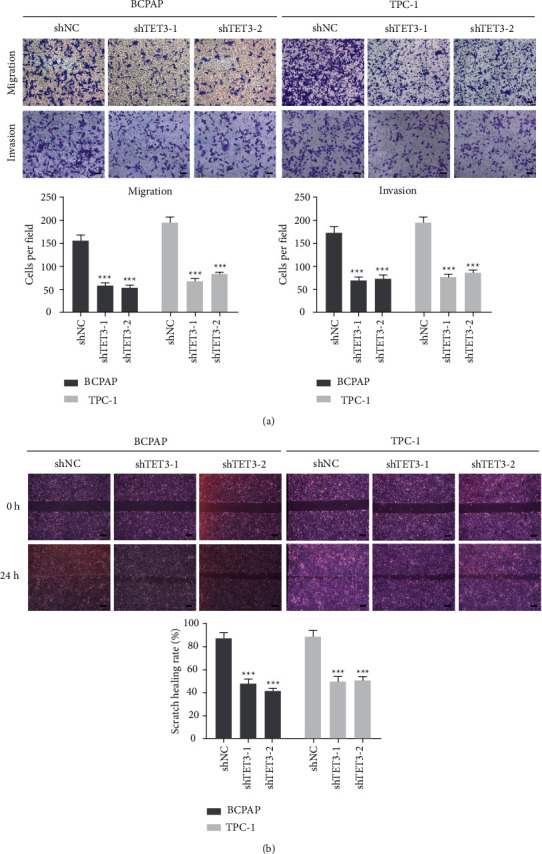
(a) The results of transwell tumor migration test showed that inhibition of TET3 expression significantly reduced the invasion ability of BCPAP and TPC-1 fine cell lines; (b) Representative images of wound-healing assays using BCPAP and TPC-1 cells showed that inhibition of TET3 expression significantly reduced the migration ability. Scale bars: 25 *μ*m. The plots below show the quantification of wound-healing assays. Data are shown as mean ± s.d. (*n* ≥ 3).

The cell growth is often suppressed by the arrested cancer cycle progression. We examined the impact of TET3 inhibition on cell-cycle progression of BCPAP and TPC-1 cells. The results demonstrated that TET3 silencing significantly increased the ratio of G1-phase cells, which implied an effect of G1/S arrest (Figures 3(a) and 3(b)). These results suggest that cell growth inhibition resulted from delayed cell-cycle progression. We performed transwell assay and wound-healing assay to detect the migration and invasion ability of PTC cells after TET3 silencing. The results showed TET3 knockdown significantly attenuated the migration and invasion of PTC cells (Figures 4(a) and 4(b)).

These results suggested that TET3 could affect the demethylation status, proliferation, and cell cycle of papillary thyroid carcinoma cells.

### 3.3. TET3 Regulates Tumorigenesis and Autophagy through AMPK Signal Pathway

According to the results of ChIP-seq in previous literature, 5hmc regulated by TET3 can inhibit AMPK signal pathway [29, 34]. AMPK is involved in the occurrence and development of a variety of tumors and can regulate tumor autophagy [25]. Therefore, we further explore whether AMPK signaling pathway plays an important role in the molecular regulation of TET3 in PTC cell tumorigenesis.

**Figure 5:**
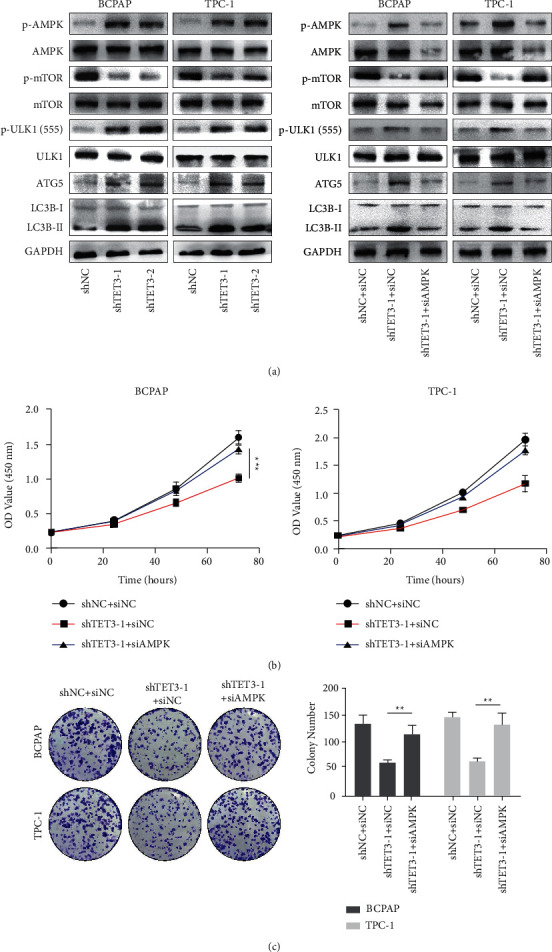
(a) Western blotting was used to detect phosphorylated AMPK, phosphorylated mTOR, phosphorylated ULK1, ATG5, and LC3BII/I in BCPAP and TPC-1 cells treated with TET3 knockdown or AMPK knockdown. CCK-8 assay (b) and colony formation assay (c) showed that AMPK knockdown partially attenuated the inhibited proliferation induced by TET3 knockdown in BCPAP and TPC-1 cells.

When TET3 was suppressed, the expression level of phosphorylated AMPK was increased (Figure 5(a)). Silencing of TET3 gene could also reduce the level of phosphorylation of mTOR, which is regarded as a key downstream target of AMPK signal and often involved in the process of cell growth, proliferation, and survival [35]. ULK1 is a key node of AMPK regulating autophagy [25], the level of ULK1 phosphorylation is increased after TET3 knockdown, and ATG5 and LC3BII/I were also upregulated (Figure 5(a)), indicating autophagy is activated.

To further validate that the TET3-regulated AMPK pathway could regulate autophagy and proliferation of PTC cells, we transfected siAMPK into PTC cells with TET3 knockdown to inhibit its activated AMPK pathway. The Western blot analysis showed the introduction of siAMPK could rescue the phenotype caused by TET3 knockdown, including the decreased level of p-mTOR and increased level of p-ULK1, ATG5, and LC3BII/I (Figure 5(a)). The analysis of CCK8 and clone formation also showed silencing of AMPK in PTC cells with TET3 knockdown can partially revert its attenuated proliferation, indicating the AMPK pathway regulated by TET3 can promote autophagy and inhibit proliferation in PTC cells (Figures 5(b) and 5(c)).

**Figure 6:**
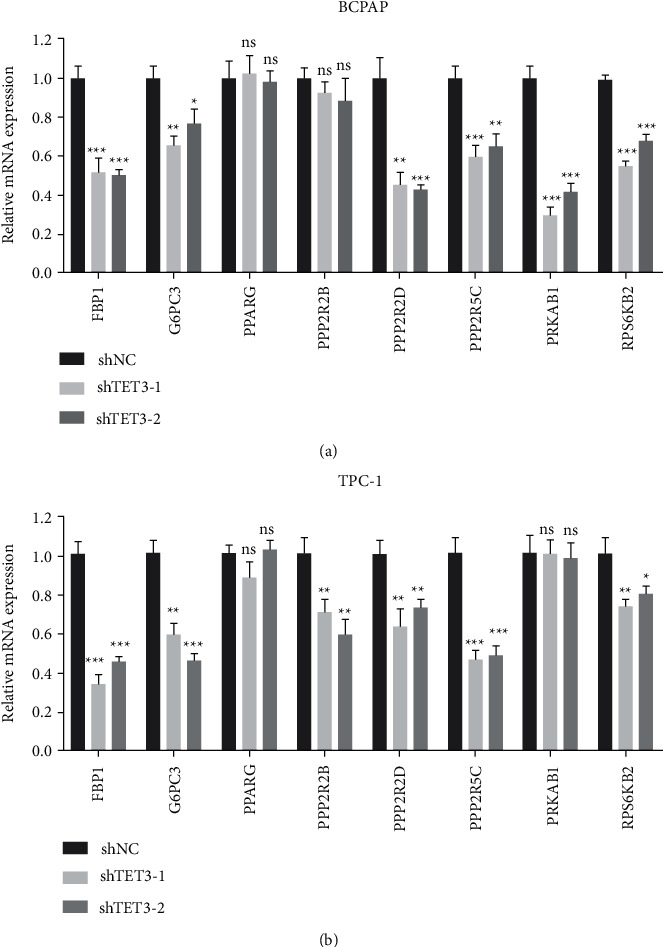
(a) and (b) qPCR was used to measure the mRNA expression levels of some AMPK pathway-related genes in PTC cells with or without TET3 knockdown.

Finally, we used qPCR to detect the expression level of several AMPK-related genes expression in PTC cells with or without TET3 knockdown. These genes were all 5hmC-upregulated genes in our previous ChIP-seq result. The results showed TET3 knockdown can significantly attenuate the transcription of some genes of them, including *FBP1*, *G6PC3*, *PPP2R2G*, *PPP2R2D*, *PPP2R5C*, *PRKAB1*, and *RPS6KB2*, suggesting these genes may have a role in TET-5hmC-regulated AMPK pathway (Figures 6(a) and 6(b)).

## 4. Discussion

The newly discovered TET family members catalyze the conversion of 5mC into 5hmC, greatly promoting DNA epigenetic modification. In the present study, we get the following results. Firstly, the content of 5hmC and the expression of TET3 in PTC tissues were significantly higher than those in normal paracancerous tissues; secondly, inhibition of TET3 expression could significantly reduce the methylation level, proliferation, migration, and invasion ability of PTC cells; thirdly, when TET3 was inhibited, the expression level of phosphorylated AMPK increased and the phosphorylation level of downstream target mTOR decreased, while the introduction of siAMPK could reverse the phenotypic changes caused by TET3 gene knockout, including the decrease of p-mTOR level. And TET3 gene knockout could enhance the inhibitory effect of AMPK on the proliferation of PTC cells, suggesting that the TET3-regulated AMPK pathway may inhibit the proliferation of PTC cells.

Previous studies have shown that TET3 protein can interact with thyroid hormone receptors (TRs): it increases the half-life of TRs by reducing ubiquitination and degradation, it stabilizes TRs' presence on chromatin, and it increases TR*α*1 capacity to mediate transcriptional activation on ligand binding. The striking reduction of TR*α*1 protein, but not transcript levels, on TET3 knockdown or knockout nicely manifests the physiological relevance and functional significance of this interaction [36].

Thyroid hormone can promote DNA demethylation in developing tadpole brain, in part by promoting TET3 recruitment to discrete genomic regions, and by inducing genes that encode DNA demethylation enzymes [37]. Global changes of 5hmC, associated with TET alteration of TET functions, have been described as a hallmark of cancer. This dysregulation has been described in both hematological and solid tumors, including colon, liver, lung, skin (melanoma), prostate, breast, and thyroid tumors [38]. Yu et al. showed that the PTC tissues and cell lines with TET1 knockdown expressed lower levels of 5hmC, contributing to aberrant DNA methylation patterns, thus exerting a tumor-suppressive function in the BCPAP cell line. This is in keeping with an intricate network connecting TET1 to the hypomethylation and activation of cancer-specific oncogenic pathways, including PI3K, EGFR, and PDGF [39]. However, we found the content of 5hmC and the expression of TET3 in PTC tissues were significantly higher in the present study. Consistently, TET3 was upregulated in ovarian cancer tissues compared with normal controls. Higher TET3 is correlated with higher stage and poor clinicopathological features [40]. Lipopolysaccharide induces the dryness of esophageal squamous cell carcinoma cells by activating the LPS-TET3-HOXB2 signal axis [41]. In this study, the expression of TET3 in PTC and its relationship with the biological behavior of cancer cells were discussed for the first time. We found that the expression of TET3 in PTC tissues was significantly higher than that in adjacent normal tissues, and inhibition of TET3 expression could significantly reduce the level of methylation and the ability of proliferation, migration, and invasion of PTC cells.

In our study, we found that when TET3 was inhibited, the AMPK signal pathway was activated, and the phosphorylation level of mTOR decreased, while the phosphorylation level of ULK1 increased. Previous studies have shown that tumor suppressor TET2 is the substrate of AMPK. AMPK phosphorylates TET2 at serine 99, thus stabilizing tumor suppressor [42, 43]. However, previous studies have shown that dexamethasone-induced inhibition of PI3-Akt pathway is mediated by TET3 [31]. TET3 promotes fibrogenic gene expression by upregulating several key TGF-*β* pathways, including TGF-*β*1, in human hepatic stellate cells [44].

Although the prognosis of patients with PTC is generally good, tumor invasiveness and metastasis are the major risk factors that lead to poor prognosis. Therefore, the major challenges in PTC research are elucidating the mechanisms underlying the invasiveness and metastasis of PTC and developing novel clinical strategies that could intervene the progression of PTC. Our study first found that the expression of TET3 is upregulated in PTC tissues, and its expression is also related to the poor prognosis of patients. TET3 can significantly promote the proliferation, invasion, and migration of PTC cells by inhibiting AMPK pathway. These findings indicate a critical role for the TET3 in the tumorigenesis and invasiveness of PTC and provide a preclinical rationale for the design of novel therapeutic strategies for this target to improve the clinical outcome of patients with PTC.

## Figures and Tables

**Table 1:** Clinicopathological features of patients with papillary thyroid carcinoma (*n* = 50).

Characteristics	Description
Gender	
Male	11 (22%)
Female	39 (78%)

Age	
≥45 years	15 (30%)
＜45 years	35 (70%)
Microcancer	9 (18%)

Lateral	
Unilateral	36 (72%)
Bilateral	14 (28%)

Focus	
Unifocal	27 (54%)
Multifocal	23 (46%)

Extraglandular invasion	
Yes	49 (98%)
No	1 (2%)

Lymph node metastasis	
Yes	41 (82%)
No	9 (18%)

Distant metastasis	0

**Table 2:** Statistics of hydroxymethylation 5hmc staining results (*n* = 50).

Name	Papillary thyroid carcinoma	Normal tissue adjacent to cancer
Size of sample	50	50

5hmc staining		
Positive	32	12
Negative	18	28

The results between the two groups were tested by chi-square test (*P* < 0.01), and the difference between the two groups was statistically significant.

**Table 3:** Relationship between 5hmc positive and clinicopathological factors in patients with papillary thyroid carcinoma (*n* = 50).

Characteristics	Amount	5hmc staining		*P* value
		Positive	Negative	
Gender				
Male	11	6	5	0.38
Female	39	26	13	

Age				
≥45 years	15	9	6	0.65
＜45 years	35	23	12	

Microcancer				
Yes	9	1	8	0.03
No	41	31	10	

Lateral				
Unilateral	36	24	12	0.58
Bilateral	14	8	6	

Focus number				
Unifocal	27	10	17	0.04
Multifocal	23	22	1	

Extraglandular invasion				
Yes	49	32	17	0.00
No	1	0	1	

Lymph node metastasis				
Yes	41	31	10	0.01
No	9	1	8	

## Data Availability

The data used to support the findings of this study are available from the corresponding author upon request.

## Supplementary Materials

Supplementary Materials Supplementary Figure 1: (A) Real-time fluorescence quantitative PCR was used to detect the expression of TET2 and TET3 mRNA in papillary thyroid carcinoma and normal thyroid tissues. Supplementary Table 1: The primer sequences are listed in Supplementary Table 1.
