# Supplementary material for: TET3 Mediates 5hmC Level and Promotes Tumorigenesis by Activating AMPK Pathway in Papillary Thyroid Cancer
Source: Int J Endocrinol. 2022 Jun 15;2022:2658727. doi: 10.1155/2022/2658727 (PMC9217609; doi:10.1155/2022/2658727)
Supplement: Supplementary Materials — Supplementary Figure 1: (A) Real-time fluorescence quantitative PCR was used to detect the expression of TET2 and TET3 mRNA in papillary thyroid carcinoma and normal thyroid tissues. Supplementary Table 1: The primer sequences are listed in Supplementary Table 1. [file 2658727.f1.zip › 2658727.f1/Supplementary Figure .docx]

**Supplementary Figure 1**


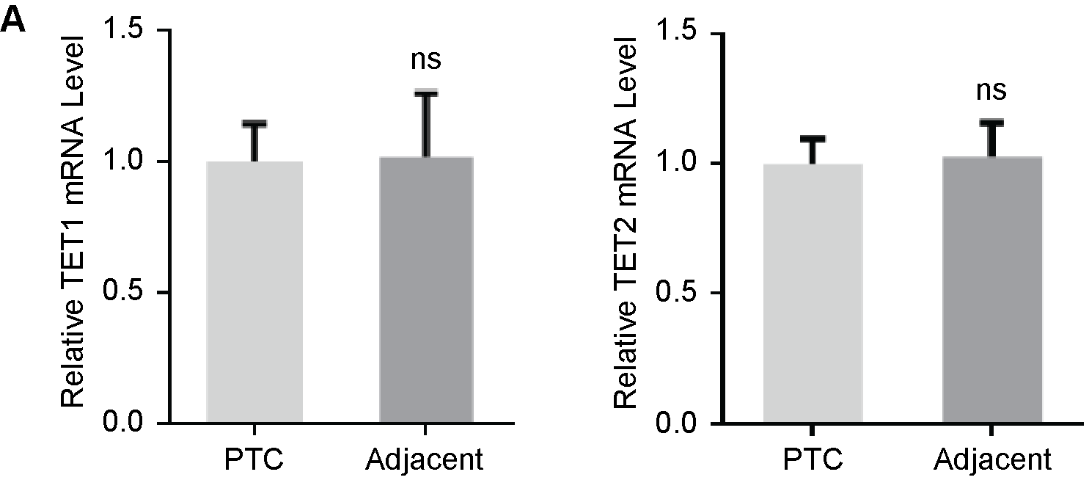


Supplementary Fig.1 (A) Real-time fluorescence quantitative PCR was used to detect the expression of TET2 and TET3 mRNA in papillary thyroid carcinoma and normal thyroid tissues.
