# Supplementary material for: TET3 Mediates 5hmC Level and Promotes Tumorigenesis by Activating AMPK Pathway in Papillary Thyroid Cancer
Source: Int J Endocrinol. 2022 Jun 15;2022:2658727. doi: 10.1155/2022/2658727 (PMC9217609; doi:10.1155/2022/2658727)
Supplement: Supplementary Materials — Supplementary Figure 1: (A) Real-time fluorescence quantitative PCR was used to detect the expression of TET2 and TET3 mRNA in papillary thyroid carcinoma and normal thyroid tissues. Supplementary Table 1: The primer sequences are listed in Supplementary Table 1. [file 2658727.f1.zip › 2658727.f1/Supplementary Table 1 (1).docx]

|  | Forward Primer | Forward Primer |
| --- | --- | --- |
| β-actin | 5ʹ-GCATGGGTCAGAAGGATTCCT-3’ | 5ʹ-TCGTCCCAGTTGGTGACGAT-3’ |
| TET1 | 5ʹ-CGCTACGAAGCACCTCTCTTA-3’ | 5ʹ-CTTGCATTGGAACCGAATCATTT-3’ |
| TET2 | 5ʹ-ATACCCTGTATGAAGGGAAGCC-3’ | 5ʹ-CTTACCCCGAAGTTACGTCTTTC-3’ |
| TET3 | 5ʹ-TCCAGCAACTCCTAGAACTGAG-3’ | 5ʹ-AGGCCGCTTGAATACTGACTG-3’ |
| FBP1 | 5ʹ-CTCTATGGCATTGCTGGTTCTAC-3’ | 5ʹ-GTTCCACTATGATGGCGTGTTTA-3’ |
| G6PC3 | 5ʹ-CCCAGGTTCACCAGTTCCC-3’ | 5ʹ-GCCGTCATTATGGGCCAGA-3’ |
| PPARG | 5ʹ-ACCAAAGTGCAATCAAAGTGGA-3’ | 5ʹ-ATGAGGGAGTTGGAAGGCTCT-3’ |
| PPP2R2B | 5ʹ-CCACACGGGAGAATTACTAGCG-3’ | 5ʹ-TGTATTCACCCCTACGATGAACC-3’ |
| PPP2R2D | 5ʹ-AAGACTTCGAGACCCATTTAGGA-3’ | 5ʹ-CGTGGACTCGCTTCTACCATA-3’ |
| PPP2R5C | 5ʹ-GAAGATCCTCGGGAGAGAGATT-3’ | 5ʹ-TCTGCTATGCCATTATGATGCTC-3’ |
| PRKAB1 | 5ʹ-CCTCACCAGAAGCCACAATAAC-3’ | 5ʹ-CGTCCACTGACCATCCACA-3’ |
| RPS6KB2 | 5ʹ-GTCAGTGAAGCACCCCTTTATT-3’ | 5ʹ-CAGGCCGTATCTTCCAGGA-3’ |
